# Tracking progression of aortic stenosis with echocardiography

**DOI:** 10.1186/s44156-025-00086-z

**Published:** 2025-08-04

**Authors:** Clay Sherrill, Jenna Port, Mohamad Wahoud, Ben Koethe, Sharanya Mohanty, Brian C. Downey, Ayan Patel, Benjamin S. Wessler

**Affiliations:** 1 Department of Medicine, Tufts Medical Center, 800 Washington St, Boston, MA 02111 USA; 2 Department of Medicine, Tufts Medical Center, Cardiovascular Center, 800 Washington St, Boston, MA 02111 USA; 3 Tufts Clinical and Translational Science Institute, 35 Kneeland Street, Boston, MA 02111 USA; 4 Cardiovascular Center, Predictive Analytics and Comparative Effectiveness Center (PACE), Tufts Medical Center, 800 Washington St, Boston, MA 02111 USA

**Keywords:** Aortic stenosis, Transthoracic echocardiography, Non-invasive cardiac imaging, Valve replacement

## Abstract

**Background:**

Transthoracic echocardiography (TTE) is used to assess aortic stenosis (AS) severity and track disease progression. As the field moves to study medical therapies to halt disease progression, reliable non-invasive imaging markers that are sensitive to small changes in disease progression are needed to enable efficient trial designs. The signal-to-noise ratio of commonly obtained TTE-based measures of progressive (non-severe) AS severity is unknown.

**Methods:**

This is a retrospective study of TTEs done at a tertiary referral centre (Tufts Medical Center, Boston MA). A cohort of patients with progressive AS who had two TTEs done within 30 days (in the absence of valve intervention) and a cohort of progressive AS patients with TTEs ≥ 1 year apart, also without valvular intervention, were assembled. Limits of agreement (LOA) and intraclass correlation (ICC) were calculated for aortic valve area (AVA) by continuity equation, peak velocity, and mean gradient. Cohen’s d-statistic (d) was calculated for each hemodynamic assessment and a composite marker to assess sensitivity for detecting disease progression normalised to measurement variability.

**Results:**

The reproducibility cohort included 24 patients. The progression cohort included 35 patients. The median age was 70 years (interquartile range [IQR] 13). 22 patients (37.3%) were female. In the progression cohort, the median time between TTEs was 2.2 years (IQR 3.1 years). In the reproducibility cohort, AVA LOA were -0.7 to 0.8, ICC = 0.61; peak velocity LOA were -149.0 to + 126.7, ICC = 0.29; and mean gradient LOA were -16.2 to 12.2, ICC = 0.06. The d-statistic for annualised change in AVA was -0.29, the d-statistic for annualised change in maximum velocity was 0.46, the d-statistic for mean gradient was 0.55. The d-statistic for a composite, including all three hemodynamic markers, was 0.45.

**Conclusions:**

Standard TTE markers of AS severity have variable sensitivity for detecting AS progression. For patients with progressive (non-severe) AS, mean gradient has the highest signal-to-noise ratio and may be the most reliable TTE-based assessment of disease progression.

**Supplementary Information:**

The online version contains supplementary material available at 10.1186/s44156-025-00086-z.

## Background

Aortic stenosis (AS) is a common progressive disease that causes an estimated 102,700 deaths globally each year [1]. While most interventional studies have focused on severe AS, there is now substantial interest in studying medical therapies to halt disease progression [2, 3]. Since progressive AS is characterised by a long progressive asymptomatic phase, studies of medical therapies will require imaging endpoints that are sensitive to disease progression. While transthoracic echocardiography (TTE) parameters are attractive imaging endpoints, the reproducibility and sensitivity of TTE-based metrics to detect early AS disease progression remains unknown. In this study, we explore the signal-to-noise ratio (defined as the magnitude of true change relative to the background measurement variability) of common TTE-based hemodynamic markers of AS to identify the imaging endpoint that is most sensitive for detecting progression of non-severe AS.

TTE remains the gold standard for tracking progression of AS [4] and accurate assessment requires interpretation of several anatomic (aortic valve area) and hemodynamic (peak velocity, mean gradient) imaging parameters. Echocardiogram-based assessments of AS severity are ideally suited as imaging endpoints because TTE is non-invasive, widely available, and individual parameters are highly correlated with hallmark features of AS (i.e. progressive valve narrowing and blood flow obstruction). However, there is no consensus on which imaging parameter should be used in prospective trials as the relative performance of these TTE based markers for tracking disease progression is unknown [2, 5–11]. Here we assess the sensitivity of TTE-based imaging markers for detecting progression of AS to identify the most promising echocardiography-based imaging markers for future clinical trials.

## Methods

### Study population

This was a retrospective cohort study of progressive (mild to moderate) AS transthoracic TTEs done at a single tertiary referral centre (Tufts Medical Center, Boston MA). Progressive AS was defined as mild to moderate AS in a manner consistent with current valve guidelines [2]. Patients in this study were divided into two cohorts: *reproducibility* and *progression* cohorts.

### Scan-scan reproducibility

The *reproducibility cohort* consisted of a convenience sample of patients with progressive AS with two TTEs done within 30 days in the absence of valve intervention. All TTEs were completed by sonographers at Tufts Medical Center.

### AS progression

The progression cohort consisted of patients with mild or mild-to-moderate AS who underwent TTE ≥ 1 year apart in the absence of valve intervention. All TTEs were performed by sonographers at Tufts Medical Center. AS progression was visualised using a linear mixed-effects (LME) model based on the sequential TTEs from the same patient [12].

### AS metrics

AS was assessed in standard integrative fashion consistent with current imaging guidelines [4, 13] by a board-certified cardiologist. The candidate imaging endpoints included standard hemodynamic assessments of valve function. Peak jet velocity is measured using continuous-wave (CW) Doppler ultrasound from multiple acoustic windows and is defined as the highest velocity signal obtained from any window. Mean pressure gradient between the left ventricle and aorta is calculated as the average CW pressure gradient across the valve throughout systole, measured from a single representative beat with optimal signal quality. Transaortic pressure is calculated using the simplified Bernoulli equation as ΔP = 4v^2^. Aortic valve area (AVA) is calculated in standard fashion using peak CW velocity. As a reference, LVOT diameter was measured in standard fashion in the parasternal long axis at the level of cusp insertion, inner-edge to inner-edge in mid-systole when LVOT is at a maximum.

### Statistical analysis

Continuous variables are expressed as median (interquartile range [IQR]). Categorical data are presented as n (%).

#### Reproducibility

For each candidate imaging variable (aortic valve area, mean gradient, peak velocity) reproducibility was assessed using Bland–Altman analysis and intraclass correlation (ICC). Limits of agreement (LOA) were calculated.

#### Rates of progression

For each imaging parameter, progression rate was determined from the *progression cohort* imaging. Progression rates are presented as annualised rates.

#### Sensitivity for detecting progression

To assess the ability for various imaging parameters to detect disease progression, we calculated the Cohen’s *d*-statistic (*d*) for each echocardiographic assessment (peak velocity, mean gradient, and AVA). This was done in a manner consistent with prior work [14]. *d* expresses measures of progression normalised by the measurement error. In this study, *d* was calculated by dividing the magnitude of the annualised progression by the measurement repeatability, defined as (1/√2) of the standard deviation (SD) of the differences between measurements at scan and rescan within the reproducibility cohort. *d* was calculated for each hemodynamic metric, as well as a composite hemodynamic marker (as seen in the *reproducibility cohort*) [14].

## Results

### Baseline assessment/characteristics

The average age of patients in the progression cohort was 67.5 ± 8.9 years and the average age in the reproducibility cohort was 73.8 ± 7.7 years. The progression cohort was 71% male while the reproducibility cohort was 50% male. Rates of comorbid illnesses are shown in Table 1.

**Table 1:** Comparison of baseline characteristics of patients in progression and reproducibility cohorts

	Progression (n = 35)	Reproducibility (n = 24)
Age (mean years ± SD)	67.5 ± 8.9	73.8 ± 7.7
Male (n, %)	25 (71%)	12 (50%)
BMI	29.7 ± 7.8	28.6 ± 5.7
Race		
White	31 (89%)	22 (92%)
Black	1 (3%)	1 (4%)
Asian	3 (8%)	1 (4%)
Comorbidities		
CHF	12 (34%)	12 (50%)
CAD	18 (51%)	14 (58%)
HTN	21 (60%)	16 (67%)
HLD	18 (51%)	16 (67%)
DM	7 (20%)	10 (41%)
Smoking	15 (43%)	12 (50%)
TTE Data (mean ± SD)		
LVEF (%)	54.9 ± 11.3	50.8 ± 14.5
AVA-1 (cm^2^)	1.3 ± 0.27	1.5 ± 0.4
AVA-2 (cm^2^)	1.1 ± 0.44	1.5 ± 0.4
Peak Velocity-1 (m/s)	2.6 ± 0.41	2.4 ± 0.53
Peak Velocity-2 (m/s)	3.12 ± 0.77	2.36 ± 0.37
Mean Gradient-1 (mm Hg)	14.9 ± 5.8	11.9 ± 5.2
Mean Gradient-2 (mm Hg)	22.9 ± 12.7	11.8 ± 5.1

*AVA* aortic valve area, *BMI* body mass index, *CAD* coronary artery disease, *CHF* congestive heart failure, *DM* diabetes mellitus, *HLD* hyperlipidaemia, *HTN* hypertension, *LVEF* left ventricular ejection fraction, *SD* standard deviation

### Reproducibility cohort

The reproducibility cohort included 24 patients with progressive AS. Baseline median AVA was 1.4 cm^2^ (IQR 0.6), median baseline peak velocity 2.4 m/s (IQR 0.6), median baseline mean AV gradient was 11.8 mmHg (IQR 6.3). This is shown in Table 2.

**Table 2:** Reproducibility of echocardiography assessments of aortic stenosis

	First TTE, median (IQR)	Second TTE, median (IQR)	Limits of Agreement (LOA)	Intraclass Correlation Coefficient (ICC)
AVA (cm^2)^	1.4 (0.6)	1.5 (0.7)	− 0.7 to 0.8	0.61
Peak Velocity (m/s)	2.4 (0.6)	2.4 (0.6)	− 149.0 to 126.7	0.29
Mean Gradient (mm Hg)	11.8 (6.3)	10.8 (9.4)	− 16.2 to 12.2	0.06

*AVA* aortic valve area, *ICC* intraclass correlation coefficient, *IQR* interquartile range, *LOA* limits of agreement, *TTE* transthoracic echocardiogram

### Progression cohort

The progression cohort included 35 patients with progressive AS. 34 patients had complete follow-up imaging for all variables of interest. The median baseline AVA was 1.3 cm^2^ (IQR 0.4), median baseline peak velocity 2.57 m/s (IQR 0.36), median baseline mean AV gradient was 13 mmHg (IQR 5.2). The median time between progression TTEs was 2.2 years (IQR 3.1 years) (Table 3).

**Table 3:** Disease progression on echocardiography

	Progression Cohort (n = 35)
Baseline TTE, median (IQR)	
AVA (cm^2^)	1.3 (0.4)
Peak Velocity (m/s)	2.6 (0.4)
Mean Gradient (mm Hg)	13 (5.2)
Follow-up TTE, median (IQR)	
∆ AVA (cm^2^/year)	− 0.06 (0.17)
Cohen’s d-statistic	− 0.29
∆ Peak Velocity (m/s/year)	0.17 (0.36)
Cohen’s d-statistic	0.46
∆ Mean Gradient (mm Hg/year)	2.23 (5.1)
Cohen’s d-statistic	0.55
Composite Metric	
Cohen’s d-statistic	0.45

*AVA* aortic valve area, *IQR* interquartile range, *TTE* transthoracic echocardiogram

### Scan-scan reproducibility

**Fig. 1:**
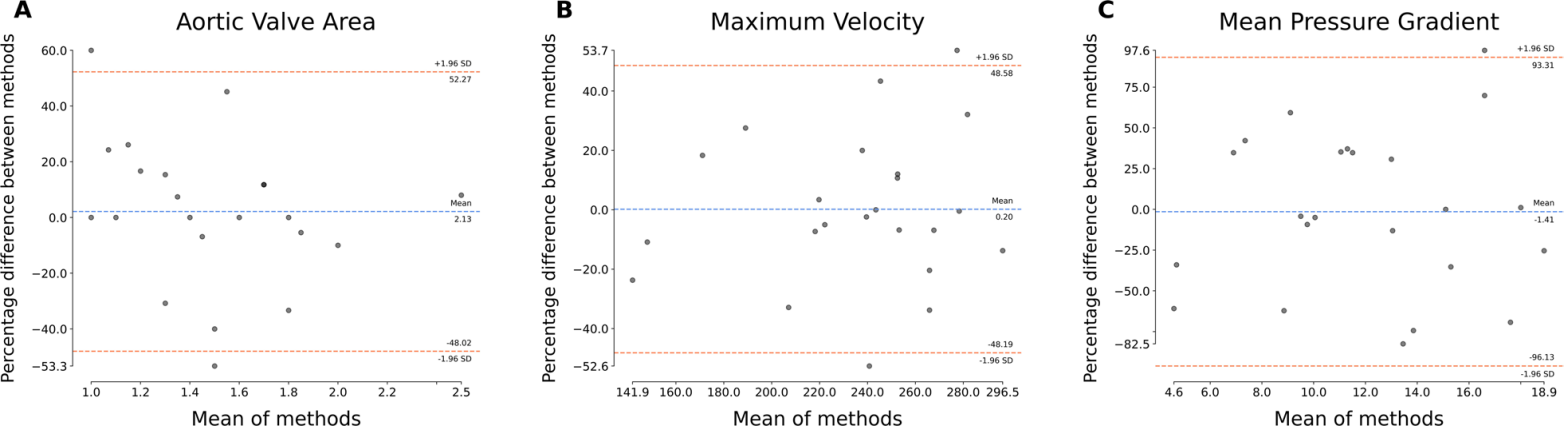
Bland–Altman plots for TTE-based imaging markers of AS severity. Subplots represent (**A**) aortic valve area, (**B**) maximum velocity, and (**C**) mean pressure gradient

For each imaging metric, Bland–Altman plots are shown in Fig. 1**.** For AVA, LOA were −0.7 to 0.8 and ICC 0.61. For peak velocity LOA −149.0 to 126.7 and ICC 0.29. For mean AV gradient LOA −16.2 to 12.2 and ICC 0.06. In contrast, the measured LVOT diameter was highly reproducible in the reproducibility cohort (Supp. Figure 1).

### Progression rates

**Fig. 2:**
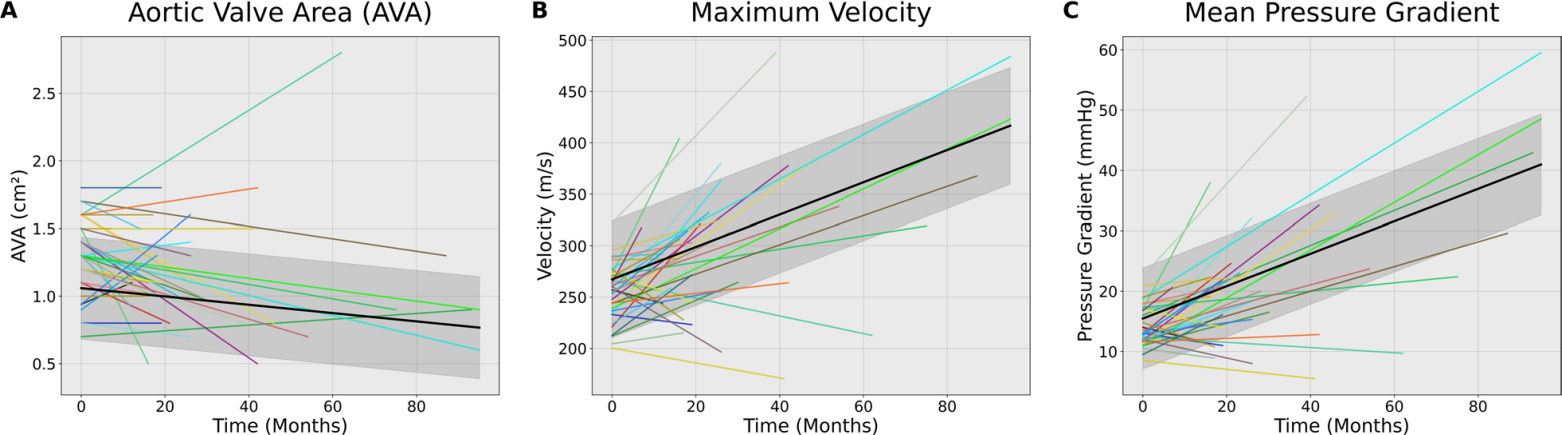
Progression of Aortic Valve Stenosis. Subplots represent (**A**) aortic valve area, (**B**) maximum velocity, and (**C**) mean pressure gradient. Each uniquely coloured line represents an individual patient, maintained across all three subplots. Progression of AS seerity from baseline as represented in a “spaghetti plot” of aortic stenosis. The time of the first TTE is considered zero, and the follow-up TTE is plotted accordingly by months since time zero. The solid black line is the overall progression fitted by a linear mixed-effects model, with the shadowed area representing the 95% confidence interval

The progression rates of change are listed in Table 3. The median annualised change in AVA was −0.06 cm^2^/year (IQR 0.17). The median annualised change in peak velocity was 0.17 m/s/year (IQR 0.36). The median annualized change in mean AV gradient was 2.23 mm Hg/year (IQR 5.10). Figure 2 graphs the respective metric for each patient over time, in addition to plotting the LME of these changes with 95% confidence intervals.

### Sensitivity for detecting progression of AS

The d-statistic for annualised change in AVA was −0.29, the d-statistic for annual change in peak velocity was 0.46, the d-statistic for mean gradient was 0.55. The d-statistic for a composite including all three hemodynamic markers was 0.45. These findings are represented in Table 3. A sensitivity analysis removing one patient that developed significant mitral regurgitation does not impact these results (Supp. Table 1).

## Discussion

The primary finding from this study is that the mean pressure gradient is the most sensitive imaging marker for detecting disease progression in patients with AS. In our cohort, it outperformed both AVA and peak AV velocity in identifying progression. Given the close association between mean pressure gradient and degree of valvular stenosis, this marker should be prioritized as a TTE-based imaging endpoint in studies of medical therapies for progressive AS.

One of the hallmark features of AS is a long asymptomatic phase dominated by fibrotic and inflammatory pathways that drive progressive valve dysfunction, restricted motion, and obstructed blood flow. TTE is the preferred imaging method for following AS progression because it is widely available and TTE-derived metrics reflect obstruction to blood flow. While TTE is widely used, there are well recognised limitations to TTE-based imaging markers. Measurement of peak aortic jet velocity is dependent upon proper alignment of the Doppler beam, and thus measurements are plane-dependent and require multiple acoustic windows to determine the highest aortic jet velocity [4]. Peak velocity is also flow-dependent and changes in cardiac output will impact apparent peak velocity. AVA as determined by the continuity equation and measured in cm^2^ is vulnerable to errors in any of the variables in this formula [4] and limitations of using this metric are increasingly appreciated [15, 16]. Left ventricular outflow tract diameter is the greatest source of error in calculation of AVA because the radius is squared in calculations. These limitations have led to a lack of consensus about which parameters are best for tracking disease progression. While the reproducibility of these measurements seem similar (based on ICC and LOA), there are apparent differences in the progression signal when corrected for measurement error.

In recent trials of medical therapies for AS, there is no consensus on which imaging parameter should be prioritised [2, 5–11]. To our knowledge, this study is the first assessment of the sensitivity of these imaging parameters for progressive (non-severe) AS. In this study, mean gradient appears most sensitive for detecting disease progression. Mean gradient reflects the pressure difference between the left ventricle and aorta and instantaneous pressures are averaged throughout the ejection period to calculate a mean pressure gradient across the valve. One reason that mean gradient is more reproducible and more sensitive to disease progression is that it does not rely on complex calculations (avoids assumptions and errors), nor does it rely on a single point measurement (peak velocity) that might be imprecise. Mean gradient requires assessment of velocity through all of systole, so measurement error at any one point has minimal effect on the overall reproducibility of the measure. Mean gradient is also less dependent on gain settings that, in the case of peak velocity, might lead to erroneous measurement of fine linear signals due to transit-time effects found at the peak of the curve. One potential limitation of mean gradient is that it may may underestimate disease severity in patients with reduced stroke volume and subsequent low-flow states [17]. Despite this limitation, mean gradient appears to be a reliable measure for serial assessment of AS in most patients.

Alternatives to echocardiography, including computed tomography aortic valve calcium (CT-AVC) scoring, have been proposed for tracking disease progression of aortic stenosis. Studies have shown CT-AVC to have high scan-scan reproducibility and demonstrate greater changes over time in comparison to traditional hemodynamic parameters used in transthoracic echocardiography [14]. Despite these advantages, CT-AVC scoring possesses significant limitations for use in clinical trials. First, CT parameters have not been validated in early progressive AS where the calcium burden is lower and may offer a less reliable signal of disease severity and progression. Additionally, CT imaging is high cost (especially compared to TTE) and has radiation exposure and requires a level of expertise not readily available at most institutions. Moreover, CT imaging is not part of routine imaging for patients with progressive AS. These limitations raise significant concerns about using CT-AVC as an imaging endpoint and support consideration of an easily accessible TTE-based imaging measure.

There are some limitations of this study that should be mentioned: the primary limitation was its relatively small sample size. The reproducibility cohort was established based on retrospective chart review, and the reasons for repeat imaging (including changes in loading conditions) were not selected. While most AS imaging in our lab includes Doppler interrogation of non-apical windows, it is possible peak values were obtained from different imaging planes at different timepoints. Additionally, the study population was racially homogenous (the progression cohort was 89% white, and the reproducibility cohort was 92% white), a concern that plagues many studies of AS. The image quality of TTE studies was not quantified, which may affect reproducibility in patients with poor windows. Lastly, all TTE-based measures of aortic stenosis severity (AVA, peak velocity, mean gradient) were obtained from echocardiography reports and documented as part of routine care.

## Conclusion

Mean gradient on TTE has the highest signal-to-noise ratio for patients with progressive (non-severe) AS and may be the most reliable imaging marker for trials of medical therapy for AS.

## Supplementary Information

Supplementary Material 1.

## Data Availability

The data used and analyzed during the current study are available from the corresponding author on reasonable request.

## Abbreviations

AS: Aortic stenosis
AV: Aortic valve
AVA: Aortic valve area
AVC: Aortic valve calcifications
CT: Computed tomography
CW: Continuous wave
ICC: Intraclass correlation
IQR: Inter-quartile range
LOA: Limits of agreement
TTE: Transthoracic echocardiography
