# Supplementary material for: Tracking progression of aortic stenosis with echocardiography
Source: Echo Res Pract. 2025 Aug 4;12:19. doi: 10.1186/s44156-025-00086-z (PMC12320324; doi:10.1186/s44156-025-00086-z)
Supplement: Supplementary file 1 — Supplementary Material 1. [file 44156_2025_86_MOESM1_ESM.docx]

**Supplemental Materials**

**Supplemental Table 1: Sensitivity analysis removing a patient who developed significant mitral regurgitation.** Cohen’s *d*-statistic across all three measures did not significantly change after the removal of a single patient who developed significant mitral regurgitation.

|  | ***Reproducibility*** |  |  | ***Progression - Non-Annualized Values*** | | | ***Cohen's D*** |  | ***Progression - Annualized Values*** | | | ***Cohen's D*** |
| --- | --- | --- | --- | --- | --- | --- | --- | --- | --- | --- | --- | --- |
| **Variable** | **Mean** | **Std Dev** |  | **Mean** | **Std Dev** |  |  |  | **Mean** | **Std Dev** |  |  |
| AVA | -0.007 | 0.365 |  | -0.211 | 0.354 |  | *-0.820* |  | -0.083 | 0.179 |  | ***-0.321*** |
| Vmax | -1.878 | 62.190 |  | 55.459 | 67.827 |  | *1.261* |  | 21.081 | 28.978 |  | ***0.479*** |
| Mean Gradient | -0.105 | 6.967 |  | 8.315 | 11.593 |  | *1.688* |  | 2.805 | 3.828 |  | ***0.569*** |
| Composite - Three Measures | -4.423 | 72.203 |  | 63.562 | 78.642 |  | *1.245* |  | 23.803 | 32.421 |  | ***0.466*** |
| Valve Calcification - Standardized | 0.042 | 0.204 |  | 0.272 | 0.410 |  | *1.885* |  | 0.075 | 0.179 |  | *0.521* |
| AVA - Standardized | -0.005 | 0.228 |  | -0.132 | 0.221 |  | *-0.820* |  | -0.052 | 0.112 |  | *-0.321* |
| Vmax - Standardized | -0.006 | 0.207 |  | 0.185 | 0.226 |  | *1.261* |  | 0.070 | 0.097 |  | *0.479* |
| Mean Gradient - Standardized | -0.002 | 0.127 |  | 0.151 | 0.211 |  | *1.688* |  | 0.051 | 0.070 |  | *0.569* |
| Composite - Three Measures - Standardized | -0.018 | 0.459 |  | 0.468 | 0.583 |  | *1.442* |  | 0.173 | 0.232 |  | *0.533* |


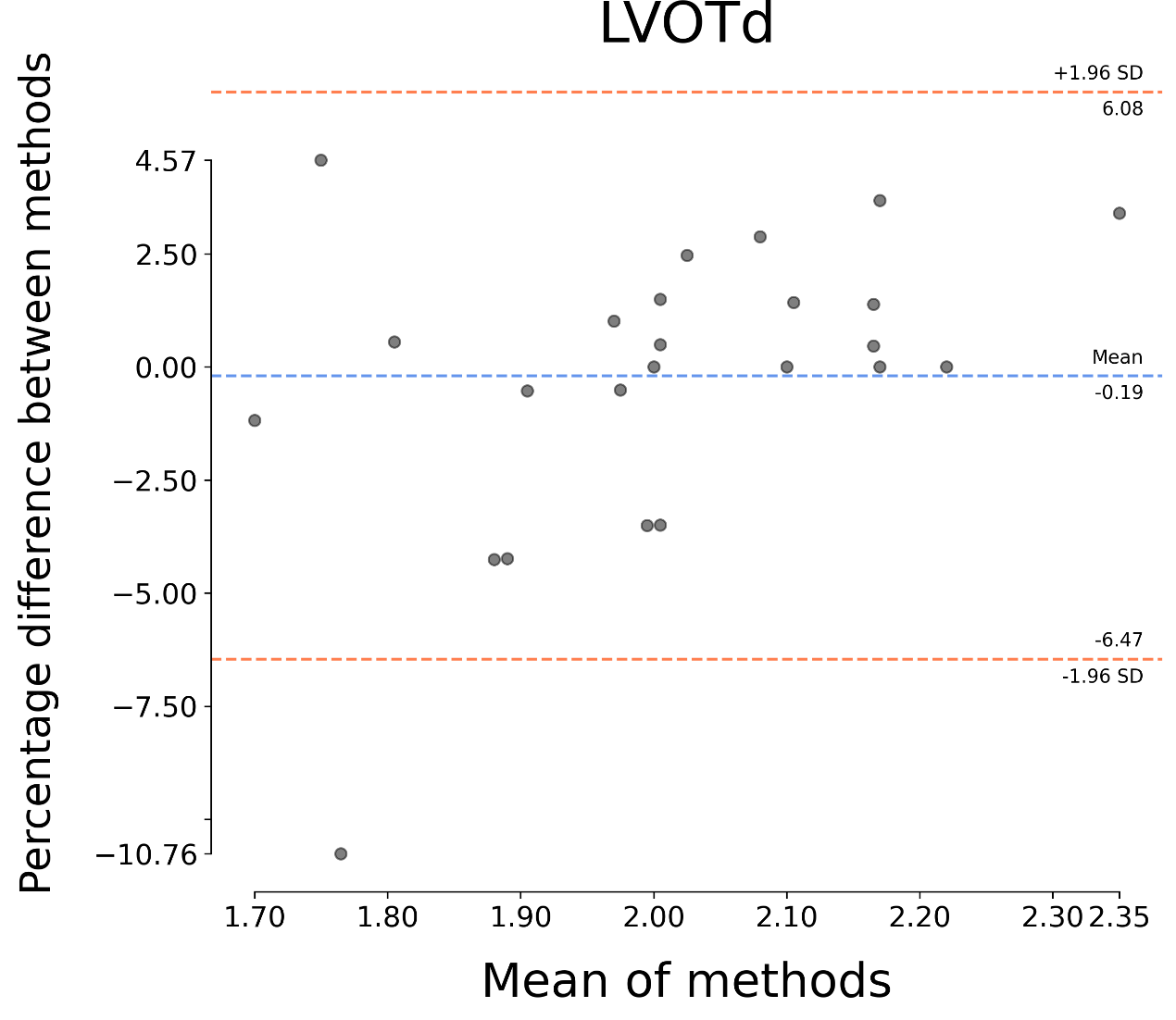
**Supplemental Figure 1: Bland-Altman plot for left ventricular outflow tract diameter (LVOTd) performed in the reproducibility cohort.** Measurements were taken by a board-certified echocardiographer. LVOTd was shown to be highly reproducible.
